# Supplementary material for: Raman Spectroscopy Combined with Malaria Protein for Early Capture and Recognition of Broad-Spectrum Circulating Tumor Cells
Source: Int J Mol Sci. 2023 Jul 28;24(15):12072. doi: 10.3390/ijms241512072 (PMC10419290; doi:10.3390/ijms241512072)
Supplement: Supplementary file 1 [file ijms-24-12072-s001.zip › Supplementary Material.pdf]

*Supplementary Material*

## Figure legends

**Figure S1. Expression and purification of VAR2CSA DBL2, VAR2CSA DBL1-2, SpyCatcher and VAR2CSA DBL2-Fc fusion antibody.**

(A). After induction for 18 h, the protein VAR2CSA DBL2, VAR2CSA DBL1-2, and SpyCatcher were purified by Ni affinity chromatography. VAR2CSA DBL2-Fc fusion antibody was purified by Protein G affinity chromatography after 3 days of protein expression. The samples were eluted with imidazole, gelled by SDS-PAGE, and stained by Coomassie brilliant blue. (B). Gel filtration chromatography analysis of purified protein. The protein was prepared at 5 mg/ml concentration in PBS buffer. Approximately 100 μl of the sample was injected onto the column and analyzed by gel filtration chromatography.

**Figure S2. The binding affinity of different concentrations of VAR2CSA DBL1-2 on A549 mixed with mouse leukocytes was detected by flow cytometry.**

(A). The A549 cells were digested by trypsin. Blood was taken from mouse hearts, and after lysis the erythrocytes, the two kinds of cells were mixed in a 5:5 ratio. VAR2CSA DBL1-2 protein diluted to the appropriate concentration was added to the cells and incubated for 30 min at room temperature. VAR2CSA protein (FITC) was labeled with anti-His tag AF488 antibody, and mouse leukocytes were labeled with anti-CD45-PerCP-CY5.5 antibody and incubated at 4° C for 30 min in the dark. (B). The relative quantification of (A) was performed. The binding affinity of VAR2CSA DBL1-2 to A549 and mouse leukocytes was calculated as Q3/Q3+Q4 and Q2/Q2+Q1, respectively. (C). The cell fraction ratio of mouse leukocytes was determined by flow cytometry. Blood was obtained from mouse hearts. After lysis the erythrocytes, 250 nM of VAR2CSA DBL2 protein was added to the cells and incubated for 30 min at room temperature. T cells, granulocytes or monocytes, B cells, VAR2CSA protein, and mouse leukocytes were labeled with anti-CD3-PE, anti-CD11b-PE-Cy7, anti-CD19-APC, anti-His tag-AF488, and anti-CD45-PerCP-CY5.5 antibody, respectively, and incubated at 4°C for 30 min in the dark. (D). The relative quantification of (C) was performed.

**Figure S3. The binding affinity of malaria protein to different cells.**

(A). The binding affinity of malaria protein VAR2CSA DBL1-2 to different human and murine-derived tumor cells was detected by flow cytometry. The tumor cells were digested by trypsin. 250 nM VAR2CSA DBL1-2 protein was added to the cells and incubated for 30 min at room temperature. VAR2CSA protein (FITC) was labeled with anti-His tag AF488 antibody and incubated at 4° C for 30 min in the dark. (B). The binding affinity of different concentrations of VAR2CSA DBL2 on A549 mixed with mouse leukocytes was detected by flow cytometry. The A549 cells were digested by trypsin. Blood was taken from mouse hearts, and after lysis the erythrocytes, the two kinds of cells were mixed in a 5:5 ratio. VAR2CSA DBL2 protein diluted to the appropriate concentration was added to the cells and incubated for 30 min at room temperature. VAR2CSA protein (FITC) was labeled with anti-His tag AF488 antibody. Mouse leukocytes were labeled with anti-CD45-PerCP-CY5.5 antibody, A549 cells were labeled with DAPI, and incubated at 4°C for 30 min in the dark. (C). The relative quantification of (A) was performed. (D). The relative quantification of (B) was performed. The binding affinity of VAR2CSA DBL2 to A549 and mouse leukocytes was calculated as Q2/Q2+Q4 and Q2/Q2+Q4, respectively. **$p < 0.01$, ***$p < 0.001$ when compared to the leukocytes group.
