# Supplementary figures and images for: Raman Spectroscopy Combined with Malaria Protein for Early Capture and Recognition of Broad-Spectrum Circulating Tumor Cells
Source: Int J Mol Sci. 2023 Jul 28;24(15):12072. doi: 10.3390/ijms241512072 (PMC10419290; doi:10.3390/ijms241512072)

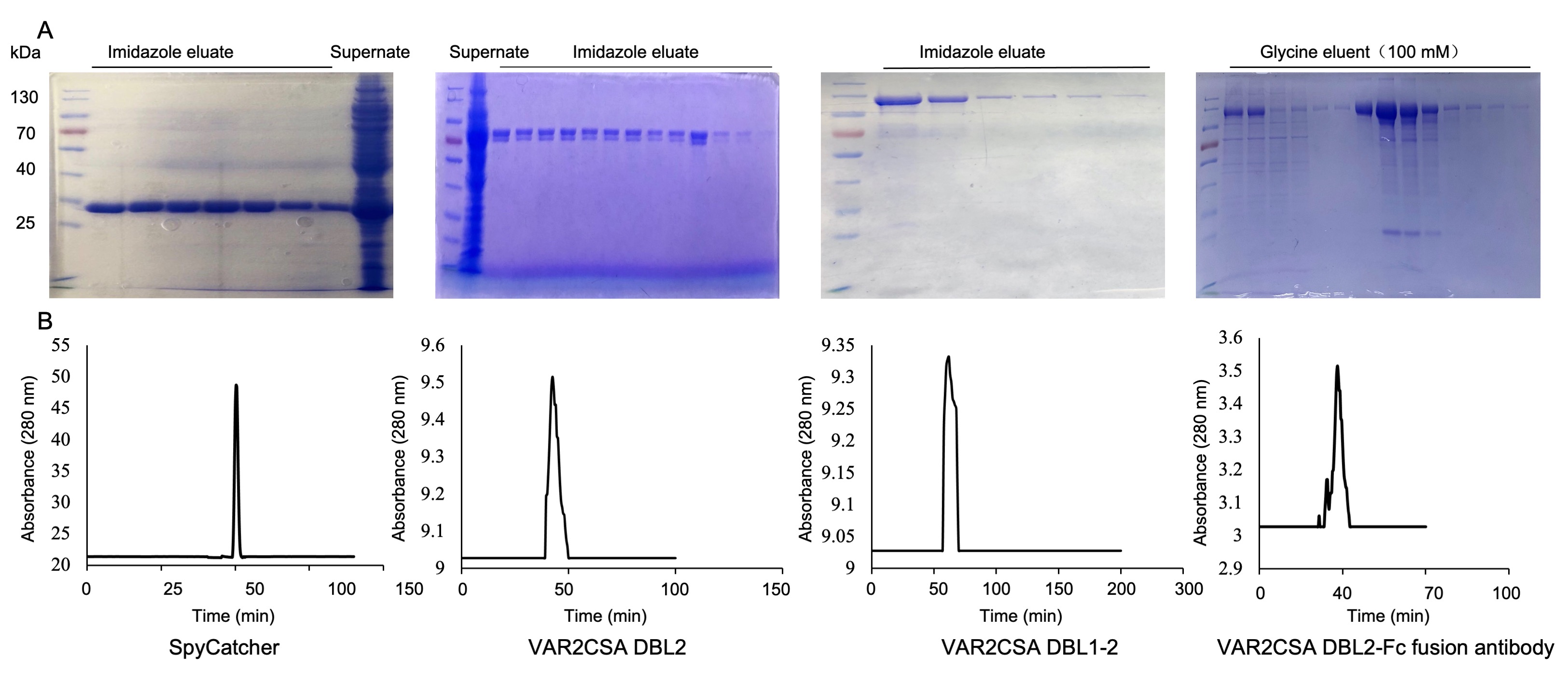

Supplement: Supplementary file 1 [file ijms-24-12072-s001.zip › Figure S1.jpg]

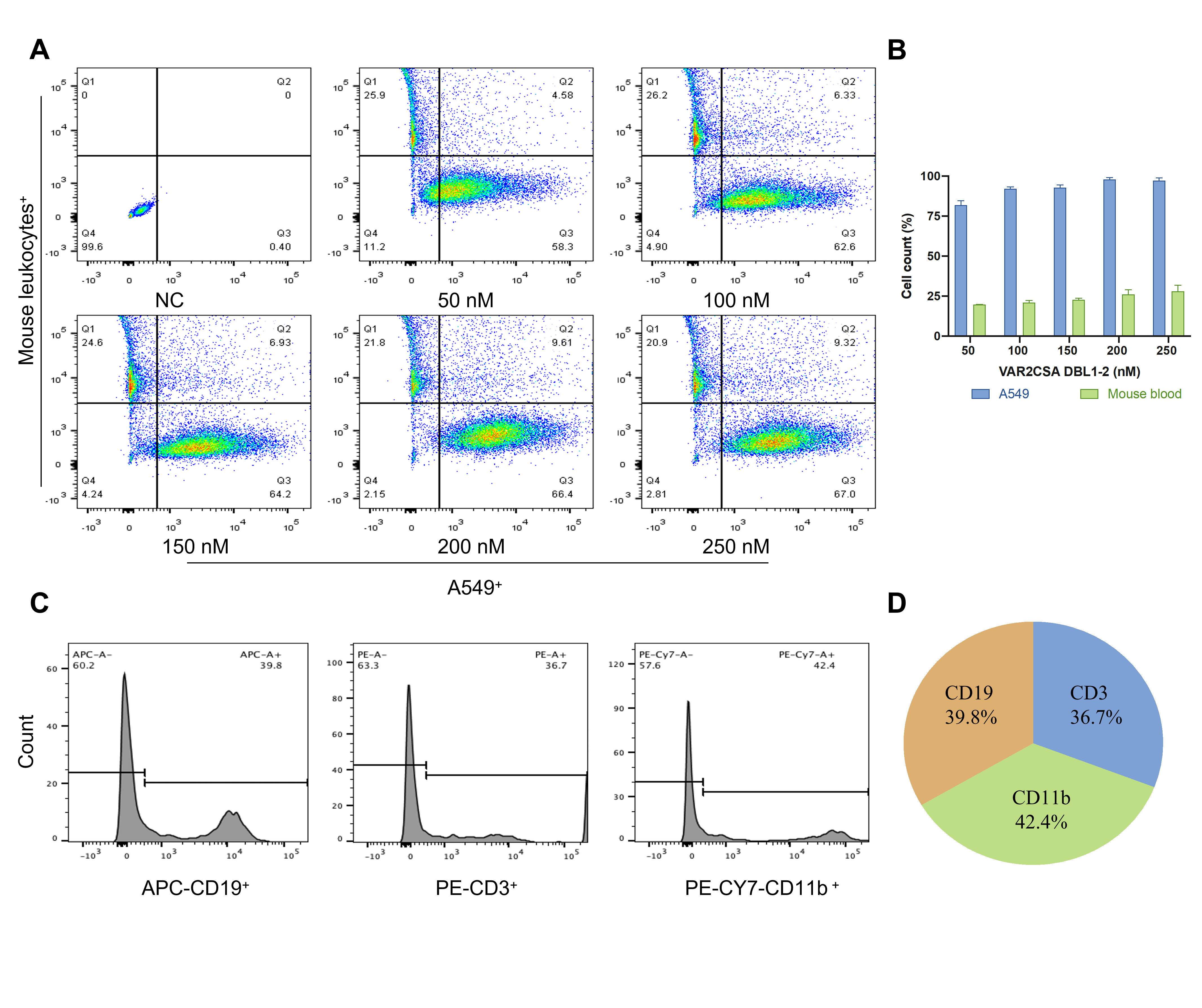

Supplement: Supplementary file 1 [file ijms-24-12072-s001.zip › Figure S2.tif]
